# Therapeutic vaccination and immunomodulation in the treatment of chronic hepatitis B: preclinical studies in the woodchuck

**DOI:** 10.1007/s00430-014-0379-5

**Published:** 2014-12-23

**Authors:** Anna D. Kosinska, Jia Liu, Mengji Lu, Michael Roggendorf

**Affiliations:** Institute for Virology, University Hospital of Essen, University of Duisburg-Essen, Virchowstrasse 179, 45122 Essen, Germany

**Keywords:** Chronic hepatitis B, Hepatitis B virus, Woodchuck hepatitis virus, Immunotherapy, Gene therapy, Therapeutic vaccination, Immunomodulation

## Abstract

Infection with hepatitis B virus (HBV) may lead to subclinical, acute or chronic hepatitis. In the prevaccination era, HBV infections were endemic due to frequent mother to child transmission in large regions of the world. However, there are still estimated 240 million chronic HBV carriers today and ca. 620,000 patients die per year due to HBV-related liver diseases. Recommended treatment of chronic hepatitis B with interferon-α and/or nucleos(t)ide analogues does not lead to satisfactory results. Induction of HBV-specific T cells by therapeutic vaccination or immunomodulation may be an innovative strategy to overcome virus persistence. Vaccination with commercially available HBV vaccines in patients with or without therapeutic reduction of viral load did not result in effective immune control of HBV infection, suggesting that combination of antiviral treatment with new formulations of therapeutic vaccines is needed. The woodchuck (*Marmota monax*) and its HBV-like woodchuck hepatitis virus are a useful preclinical animal model for developing new therapeutic approaches in chronic hepadnaviral infections. Several innovative approaches combining antiviral treatments using nucleos(t)ide analogues, with prime-boost vaccination using DNA vaccines, new hepadnaviral antigens or recombinant adenoviral vectors were tested in the woodchuck model. In this review, we summarize these encouraging results obtained with these therapeutic vaccines. In addition, we present potential innovations in immunostimulatory strategies by blocking the interaction of the inhibitory programmed death receptor 1 with its ligand in this animal model.

## Introduction

More than 240 million people worldwide are persistently infected with hepatitis B virus (HBV) and are at risk of developing chronic liver disease, cirrhosis and hepatocellular carcinoma (HCC) [1]. An effective and affordable therapy to achieve sustained suppression of HBV replication and remission of liver disease is urgently needed. Pegylated interferon-alpha 2a (IFN-a) is recommended for the treatment of chronic hepatitis B (CHB) in the current consensus guidelines of many countries. Compared with conventional recombinant IFN-a, however, pegylated IFN-a alone or in combination with nucleoside analogues does not significantly increase the rate of sustained response [2, 3]. Nucleos(t)ide analogues, such as, entecavir and tenofovir, suppress HBV replication and result in the improvement of liver architecture. However, these agents cannot eradicate HBV genomes from the liver and may further limited by the development increasingly select drug-resistant mutants with prolonged use [4, 5]. Therapy with additional antiviral drugs targeting other steps in the viral life cycle, in combination with immunomodulatory options, might be more beneficial and effective.

More than 90 % of acutely infected adults resolve clinical symptoms and maintain lifelong protective immunity by mounting a vigorous, multi-specific immune response to HBV proteins. By contrast, patients with chronic hepatitis B tend to have delayed, transient or narrowly focused T cell responses [6–8]. Patients who spontaneously recover from HBV infection might experience reactivation of HBV under immunosuppressive treatments. Thus, the specific immune responses to HBV remain crucial for the long-term control of HBV infection even after resolution of the acute infection. For chronically infected patients, immunostimulatory and immunomodulatory strategies to boost or to broaden the weak virus-specific T cell response have been proposed to reach an effective control of viral infection.

## Therapeutic vaccination studies in patients with chronic hepatitis B

Since more than 20 years, numerous clinical trials exploited the conventional prophylactic vaccine based on the hepatitis B surface antigen (HBsAg) for therapeutic vaccination (Table 1). These studies demonstrated reductions in viremia, seroconversion of the hepatitis B “e” antigen (HBeAg) to anti-HBe and HBV-specific T cell responses in some patients after vaccination. However, the antiviral effect was only transient and did not lead to an effective control of the HBV [9–17].

**Table 1:** Therapeutic vaccination studies in patients with chronic hepatitis B using the conventional HBsAg vaccine, immune complexes, T cell vaccines and combination therapy

Vaccination strategy	Vaccine	Antigen	References
Protein vaccine	Licensed HBsAg vaccine for prophylactic use	Pre-S2/S	Pol et al. [13, 14] Coullin et al. [9] Ren et al. [15] Yalcin et al. [17] Dikici et al. [11]
		Pre-S1/pre-S2/S	Jung et al. [12] Safadi et al. [16]
	Immune complexes of HBsAg–anti-HBs	S	Wen et al. [18] Yao et al. [19] Xu et al. [21] Xu et al. [22]
T cell vaccine	CTL-peptide vaccine	HBcAg	Heathcote et al. [112]
	DNA vaccine (HBsAg)	Pre-S2/S	Mancini-Bourgine et al. [23, 24]
Combination therapy	Antivirals and protein vaccine (HBsAg)	S	Dahmen et al. [113] Horiike et al. [114] Vandepapeliere et al. [115]
		Pre-S1/pre-S2/S	Hoa et al. [28]
	Antivirals and T cell vaccine	Pre-S2/S	Godon et al. [26] Fontaine et al. [25]
		Pre-S1/pre-S2/S, HBcAg, polymerase	Yoon et al. [27]

A more sophisticated therapeutic vaccination based on HBsAg complexed with human anti-HBs was proposed by the group of Wen et al. [18]. Immunogenic complexes (IC) stimulate robust T cell responses by increasing uptake of HBsAg through Fc receptors on antigen-presenting cells (APC) and, therefore, enhance HBsAg processing and presentation. It was demonstrated that this vaccine administered to HBeAg-positive patients led to decrease of HBV DNA in serum and HBeAg seroconversion in some subjects [19]. In a phase II B clinical trial, HBeAg seroconversion was observed in about 21.6 % of treated patients. Moreover, a moderate decrease in serum HBV DNA and HBsAg levels was observed after treatment [20, 21]. Very recently, a large phase III clinical trial with 12 injections of IC complex failed to show any therapeutic efficacy when compared to the placebo control injected only with alum [22]. Overstimulation with IC-based vaccine did not increase but decreased efficacy of the therapeutic vaccination. These results underline that an appropriate immunization protocol is crucial for the efficacy of therapeutic vaccination.

DNA vaccines using plasmids expressing viral proteins have gained popularity given their ability to induce strong cellular and humoral immune responses. Several phase I clinical studies investigated the therapeutic efficacy of plasmid DNA vaccines expressing HBsAg in chronic HBV carriers. These studies showed evidence for the safety of HBV DNA vaccination (for details see below), but T cell responses were restored or activated at only a low level. Furthermore, DNA vaccines expressing only HBsAg did not result in significant suppression of viremia in chronic carriers of HBV [23, 24].

From results of these studies, it can be concluded that the therapeutic vaccination alone is not sufficient to achieve the control over HBV. High load of virus particles and large amounts of HBsAg in the liver and peripheral blood may be responsible for the immune tolerant status in the patients. Therefore, pretreatment with nucleos(t)ide analogues has been proposed to achieve better CD8 T cell response and subsequent therapeutic efficacy after administration of DNA vaccines.

Recently, the results of the trial of this combination therapy have been published. In a large double-blind study, 70 patients were treated effectively with nucleos(t)ide analogues for a median of 3 years resulting in undetectable levels of HBV DNA and thereafter randomized into two groups: one received five intramuscular injections of DNA vaccine expressing HBsAg and one received placebo. Nucleos(t)ide analogues were stopped. Although this combination therapy was fairly well tolerated, the HBV DNA vaccine did not decrease the risk of relapse in HBV-treated patients and did not restore the anti-HBV immune response despite effective viral suppression by analogues [25, 26].

During a study in Korea, 27 patients randomly received either adefovir (ADV) alone or ADV in combination with HBsAg-expressing DNA vaccine. Therapeutic vaccination was safe and tolerable in CHB patients. Vaccine-induced HBV-specific T cell responses and HBeAg seroconversion were weaker in Korean patients than in Caucasian patients [27]. Asian patients, who are generally infected via vertical transmission, may have a higher level of immune tolerance than Caucasians who are usually infected later in life. Improved vaccines for breaking immune tolerance may be needed to develop effective therapeutic HBV DNA vaccines.

The aim of a study in Vietnam was to evaluate viral suppression following combined treatment with a new vaccine containing all three envelope proteins of HBV (pre-S1/pre-S2/S) and lamivudine in CHB patients. The enhanced suppression of viremia in the combination group was reversed after the discontinuation of vaccine treatment, suggesting that booster doses are required for a sustained viral response. Anti-HBs was detected in 55/120 vaccine recipients, but only three patients demonstrated HBsAg loss, indicating that the vaccine-induced anti-HBs was unable to completely neutralize HBsAg in the serum [28].

## Woodchuck model

**Fig. 1:**
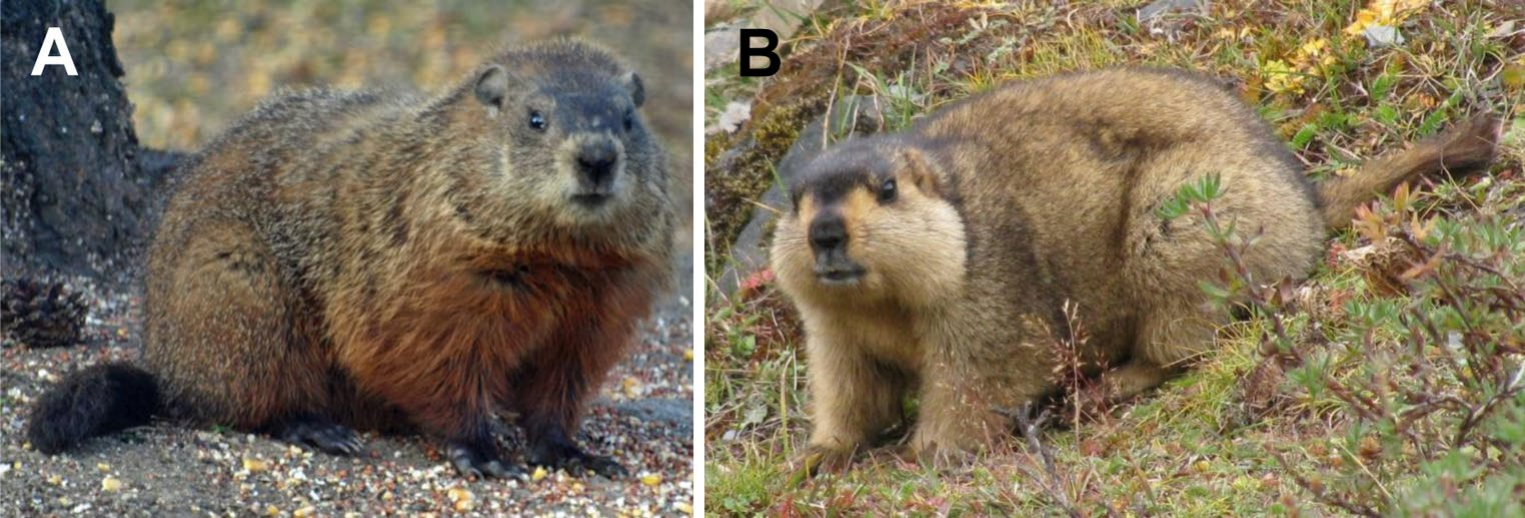
Pictures of eastern woodchuck *M. monax* (**a**) and *M. himalayana* (**b**). Himalayan marmots are closely related to the woodchucks and can be infected with WHV. They are about the size of a large housecat and live in colonies

The eastern woodchuck (*Marmota monax*) is naturally infected by woodchuck hepatitis virus (WHV) which was discovered in 1978 [29]. WHV was found to be closely related to hepatitis B virus (HBV) [30] and classified as the second member of the genus ortho-hepadnavirus, family hepadnaviridae. In contrast to HBV-associated HCC in patients without a preferred integration site of HBV DNA, a frequent integration of the WHV genome close to the N-myc and c-myc gene has been observed in woodchucks developing HCC [31]. Infections of woodchucks with WHV have been shown to be endemic in the Mid-Atlantic States of the USA, whereas in the State of New York and New England woodchucks are rarely infected with WHV. Recently, a Chinese marmot *Marmota himalayana* was found to be susceptible to WHV infection [32] (Fig. 1). These findings indicate that *M. himalayana* is phylogenetically closely related to *M. monax*. Therefore, this Chinese Marmota species can be explored as a model for hepadnaviral infection and prevention of infection [33] and in the future for the new therapies.

The molecular characterization of WHV and experimental infection of woodchucks with WHV have been of great value in modelling several aspects of hepadnaviral infection in humans, e.g. the natural course of infection [34–37], immunopathogenesis [38–42], including innate and adaptive immune responses, host and viral factors associated with development of chronicity.

From a medical point of view, the woodchuck model has been used to develop new strategies for prevention of infection [43, 44], post-exposure prophylaxis of hepatitis B and therapy of chronic hepadnaviral infection including: nucleoside analogues [45–49], non-nucleoside analogues [50], therapeutic vaccination (reviewed in [51]) and gene-therapeutic approaches for treatment of HCC [52]. Liver transplantation has recently been established for woodchucks to study early events in re-infection and adoptive immune transfer [53]. Lately, a significant progress has been made in this model to demonstrate that both cellular immune responses are needed for elimination of the virus from hepatocytes or to at least control viral replication [38, 39, 41].

This review is focusing on the characterization of woodchuck genes related to innate and adaptive response, the recent development of new tools to determine virus-specific T cell response, therapeutic vaccines, and finally immunostimulatory and immunomodulatory approaches to treat chronic WHV infection. These new findings in this preclinical model will help the development of new strategies to treat chronic HBV infection in patients.

## Cloning and characterization of components of woodchuck immune system

In recent years, many efforts have been devoted to cloning and characterization of components of the woodchuck immune system. A number of immune function-related genes including cytokines and their receptors, immune cell surface markers and other immune function-related proteins have been cloned and characterized.

So far, important woodchuck cytokines and their receptors such as TNF-α, IFN-α, IFN-γ, IL-12, IL-15, GMCSF, lymphotoxin (LT)-α and IL-10R have been cloned and tested for their biological activities [54–61]. In patients, IFN has been used in the treatment of CHB for many years. Therefore, the IFN system has also been characterized in woodchucks. Woodchuck IFN-α was shown to reduce WHV surface antigen expression in a dose-dependent fashion in WHV-infected woodchuck hepatocytes [62]. The woodchuck IFN-α/β system and their expression in peripheral blood lymphocytes (PBLs) from naïve and WHV-infected woodchucks have also been studied [63]. The woodchuck IFN-α genes could be classified into ten subtypes and three pseudotypes. Poly(I:C) stimulation on naïve woodchuck PBLs could induce IFN-α subtypes one, four and five production, indicating a selective expression of woodchuck IFN-α subtypes. Moreover, PBLs from chronically WHV-infected woodchucks showed a reduced ability to produce woodchuck IFN when stimulated with poly(I:C). The complete or partial sequences of the type I IFN receptors (IFNARs) of woodchucks were also obtained and analysed by Fan et al. [64]. IFN-α or IFN-γ stimulation significantly upregulated IFNAR2 expression in primary woodchuck hepatocytes. A decreased IFNAR1 and IFNAR2 expression was observed in woodchucks chronically infected with WHV. These data are essential for studying type I IFN-related innate immunity and therapy in hepadnaviral infection in the woodchuck model. IL-10 is a pleiotropic cytokine acting on a variety of immune cells through its cell surface receptor (IL-10R). It has been suggested to resuscitate antiviral immunity by interfering with IL-10/IL-10R pathway. An increased production of IL-10 was observed in patients with CHB [65], which hints that blockade of IL-10R might become a feasible therapeutic approach for CHB. Very recently, Jiang et al. [54] successfully cloned woodchuck IL-10R and generated antibodies against this molecule. The blockade of woodchuck IL-10R enhanced the proliferation and degranulation of specific T cells from chronically WHV-infected woodchucks in vitro. This work provides a basis for potential therapeutic approaches in chronic HBV infection.

Important woodchuck immune cell surface molecules which have been cloned so far can be divided into two categories based on their function: molecules involved in innate immunity and molecules involved in adaptive immunity.

Toll-like receptors (TLRs) are a class of molecules that play a key role in the innate immune system. Recent progress in this field revealed that there are significant interactions between the TLR system and pathogens in chronic viral infections [66]. So far, TLR2, TLR3, TLR4, TLR7, TLR8 and TLR9 have been successfully cloned in woodchucks [67]. In a recent study, Zhang et al. [66] showed that TLR2 ligands induced the activation of NF-κB, PI3K/Akt and different arms of MAPK signalling pathways and the production of pro-inflammatory cytokines in woodchuck hepatocytes. TLR2-mediated innate immune responses reduced replication and gene expression of HBV in HepG2.2.15 cells and WHV in primary woodchuck hepatocytes (see also article from Zhang and Lu, in this issue). In chronic WHV carriers woodchuck model, relatively low levels of TLR2 expression were found in PBMCs and in liver tissues. TLR2 expression in PBMCs was inversely correlated with WHV DNA titres in acute WHV infection and in entecavir-treated chronic WHV carriers.

An effective immune response against viral infections depends on the activation of CD8 T cells that can clear infection by killing virus-infected cells. Therefore, sequence information of woodchuck CD3, CD4 and CD8 has been used to determine the kinetic of the influx of T cells into the liver during incubation period and acute or chronic WHV infection. In week two post-infection, an influx of CD3+ lymphocytes could be observed and reached higher levels prior and during the recovery phase. The peak level of CD4+ and CD8+ T cells coincided with recovery. During transient infection, T cells can accumulate in the liver and reach up to two-thirds of the total number of liver cells [35]. In the adaptive immune response, CD28 and CTLA-4 are known to play important roles for the regulation of T cell activation by delivering costimulatory signals. The complete coding regions of woodchuck CD28 and cytotoxic T-lymphocyte-associated antigen 4 (CTLA-4) have been cloned and sequenced [68]. Woodchuck CD28 showed a similarity of 76 and 70 % to its human and mouse homologues, respectively, according to the deduced amino acid sequences. Woodchuck CTLA-4 has a higher similarity of 86 and 75 % to the corresponding human and mouse CTLA-4 molecules, respectively. The strict conservation of critical amino acid residues like cysteine and asparagine residues in woodchuck CD28 and CTLA-4 suggests that both molecules may structurally resemble their human or mouse homologues. A hexapeptide motif MYPPPY which has been supposed to be essential for the interaction with CD80 is present in both woodchuck CD28 and CTLA-4 [68].

The advances in sequencing technology provide new tools to characterize genes of the woodchuck immune system in large scale. Fletcher et al. [69] performed the sequencing, assembly and annotation of the woodchuck transcriptome, together with the generation of custom woodchuck microarrays. By using this new platform, they characterized the transcriptional response to persistent WHV infection and WHV-induced HCC. Liu et al. have also performed de novo woodchuck transcriptome assembly by using deep sequencing technology (unpublished data). With the help of this advanced technology, sequence information of important immune genes such as APOBEC3 of woodchucks has been revealed. It has been shown that upregulation of APOBEC3 led to specific and non-hepatotoxic degradation of nuclear HBV cccDNA [70]. Therefore, future cloning and characterizing of APOBEC3 in the woodchuck model will evaluate the therapeutic potential for CHB. In summary, these efforts on establishing the translational value of the woodchuck model can provide new insight into characterizing immune pathways which may play a role in the persistence of HBV infection.

## Evaluation of T cell response in woodchuck model

Studies in patients underline the important role of HBV-specific T cell response as a leading factor of viral clearance. For many years, the lack of appropriate methods to evaluate antigen-specific T cell responses was the serious limitation of this model. The establishment of the assays for monitoring of cellular immune response in woodchucks is of great importance for a reliable evaluation of therapeutic and immunomodulatory strategies for treatment of CHB in the woodchuck model.

Development of the 2[^3^H]-adenine-based proliferation assay enabled to detect the T-helper lymphocyte responses after stimulation of woodchuck PBMCs [39, 41]. In addition, several T-helper epitopes within WHcAg [39, 41] were identified in PBMCs from acutely WHV-infected animals. Significant progress in studying the T cell response of woodchucks was achieved by introduction of the flow cytometric CD107a degranulation assay that enables the detection of WHV-specific cytotoxic T cells (CTLs) in woodchuck PBMCs and splenocytes [38]. Several studies demonstrated that detection of CD107a, as a degranulation marker, is a suitable method for determination of antigen-specific cytotoxic T lymphocytes [71, 72].

Introduction of the immunological tools for studying of the T cell response in woodchucks revealed a significant similarity between the pathogenesis of WHV infection in woodchucks and HBV in humans. It was demonstrated that acute self-limiting and resolved WHV infections correlate with robust multifunctional T-helper and cytotoxic T cell responses, while WHV chronic carriers demonstrate weak or no virus-specific T cell responses against the viral proteins (Table 2) [38, 39, 41]. Moreover, these studies confirmed that the efficient cellular immune response to viral antigens results in liver injury and is necessary for viral clearance.

**Table 2:** Correlation between the outcome of WHV infection and cellular immune response to WHsAg, WHxAg and WHcAg in woodchucks neonatally infected with WHV: 11 woodchucks recovered from infection and 23 developed chronic hepatitis

Outcome of the infection	% of woodchucks responding to:				% of PBMC samples positive to:			
	rWHcAg	rWHsAg	rWHxAg	C97-110	rWHcAg	rWHsAg	rWHxAg	C97-110
Resolved (*n* = 11)	100 (11/11)	82 (9/11)	91 (10/11)	100 (11/11)	59 (32/54)	34 (18/53)	34 (17/50)	51 (28/55)
Chronic (*n* = 23)	39 (9/23)	22 (5/23)	26 (6/23)	17 (4/23)	6 (16/262)	2 (6/242)	4 (10/242)	2 (5/265)

The numbers of PBMC samples detected positive to WHV antigens during the study are given in brackets [41]

## Therapeutic immunization in the woodchuck model: viral vectors and prime-boost strategy

Recently described advancements in the characterization and monitoring of the woodchuck immune system during the WHV infection made this animal model particularly useful for development of the immunomodulatory approaches in CHB. The pioneer investigations with therapeutic vaccines based on WHV core [73] or surface antigens in combination with a helper peptide named FIS (encompassing amino acids 106-118: FISEAIIHVLHSR from sperm whale myoglobin) [74], or with potent Th1 adjuvants like monophosphoryl lipid A [75] did not lead to satisfactory results. Those experiments proved that vaccinations could induce specific B cell and/or T cell responses in chronic WHV carriers. However, this alone was not sufficient to achieve the control of virus replication, as the very high load of virus may be responsible for the immune tolerant status in the animals. This idea is supported by Boni et al. [76, 77] reporting that the T cell response to HBV was successfully restored in patients treated with lamivudine. In addition, the quantity of antigen particularly the WHV surface antigen (WHsAg) to which the immune system is exposed can induce different degrees of functional impairment of antiviral T cells, up to physical T cell deletion [78, 79].

Combination therapy using lamivudine and serum-derived WHsAg vaccination showed no effect on induction of anti-WHs antibodies or reduction of WHV DNA [80]. Our group evaluated the efficacy of the combination therapy in the woodchuck model by combining lamivudine treatment, DNA vaccination (three plasmids expressing WHsAg, WHcAg and woodchuck IFN-γ) and WHsAg/anti-WHs immunogenic complexes vaccination [81]. The triple combination led to a decrease in WHV viral load up to 2.9 log, in serum WHsAg up to 92 % and in development of anti-WHs antibodies. Nevertheless, these effects were not sustained and all parameters reached the baseline levels shortly after withdrawal of lamivudine treatment. In addition, the vaccination did not induce WHV-specific T cell responses in the majority of woodchucks, even in animals that exhibited virological responses. Later, we modified this protocol by using the more potent antiviral drug entecavir (ETV) and increasing the number of the immunizations (with plasmids expressing WHsAg and WHcAg from three to six) (Lu et al., unpublished results). A significant delay of the rebound of viremia was observed in woodchucks which received additional vaccination, compared to controls treated only with ETV. In another study, chronic WHV carriers received a treatment of the potent antiviral drug clevudine in combination with an alum-adsorbed WHsAg vaccine. Combination treatment resulted in significant and sustained reduction of WHV DNA loads and WHsAg concentrations in most treated animals. Compared to vaccination alone, combination treatment induced a more robust anti-WHs response [82, 83].

The results of these studies clearly showed that combination of antiviral treatment and vaccination is more effective in inducing virus-specific T cell responses than therapeutic vaccination alone. Nevertheless, the efficacy of these approaches was still too limited when applied for treatment of CHB. The vaccination strategies used in some of these studies were even not able to boost a functional antiviral T cell response. A significantly better induction of WHcAg-specific T cells using more potent vaccines, such as recombinant viral vectors, may be required to achieve sustained antiviral response and viral clearance.

Recombinant adenoviral vectors (AdV) proved to elicit a vigorous and sustained humoral and T cell responses to the transduced antigen [84, 85]. Adenoviral vectors also act as a natural adjuvants causing DC maturation, enhanced antigen presentation and secretion of antiviral cytokines, such as IFN-α, TNF-α and IL-6 [86]. However, even single immunization with recombinant adenoviruses may induce immunity, predominantly neutralizing antibodies, against the vector itself. This negative effect of the adenovirus-induced immunity against the vaccine may be overcome by heterologous prime-boost regimen. In particular, subsequent priming immunizations with plasmid DNA vaccine followed by a booster vaccination with AdV seem to be a very promising strategy. DNA prime–adenovirus boost regimen proved to induce more robust and potent immune response in comparison with plasmid DNA alone and provided protection against the pathogen challenge in several animal models of infectious diseases [87–89] (see also article from E. Barnes in this issue).

Recently, our group has investigated whether the heterologous prime-boost immunization strategy using plasmid DNA and recombinant adenoviral vectors may improve the efficacy of the therapeutic vaccination in CHB in the woodchuck model. A new DNA plasmid (pCGWHc) and an adenoviral serotype 5 vector (Ad5WHc) and a chimeric Ad5 displaying Ad35 fibre (Ad35WHc) showing high expression levels of WHcAg were constructed [90]. The increased antigen expression was achieved by insertion of an intron sequence in the expression cassette of the vaccines. Preliminary results showed that the new vaccines are able to induce strong and sustained WHcAg-specific T cell response in mice and naïve woodchucks. Interestingly, immunization with AdVs led to rapid and massive production of anti-WHs antibodies and as a result resolution of infection after the WHV challenge [90].

The DNA prime–AdV boost immunization strategy was further used as a therapeutic vaccine against chronic WHV infection in combination with antiviral treatment with ETV. Six chronically WHV-infected woodchucks were treated for 23 weeks with ETV. Starting from week eight, four of the six ETV-treated animals received subsequently nine intramuscular immunizations with: DNA plasmids expressing WHcAg (pCGWHc) and WHsAg (pWHsIm), Ad5WHc and Ad35WHc. WHsAg- and WHcAg-specific T-helper and cytotoxic T cell responses were detected in all chronic carriers that received immunizations, but not in ETV only treated animals. In addition, woodchucks receiving the combination therapy showed a prolonged suppression of WHV replication and lower WHsAg levels compared to controls. Excitingly, two of four immunized carriers remained WHV DNA negative after the end of ETV treatment and developed anti-WHs antibodies [91]. These data are encouraging and demonstrate that the combined antiviral and vaccination approach efficiently elicited sustained immunological control of chronic hepadnaviral infection in woodchucks.

## Combining therapeutic vaccination and modulation of T cell function

Persistent HBV infection is associated with functional exhaustion of virus-specific CD8 T cells [92]. This defect in virus-specific T cells is one of the primary reasons for the inability of the host to eliminate the persisting pathogen. Although it has been shown that nucleoside analogues treatment can induce the recovery of HBV-specific CTL activity in patients [76], this effect is only transient [77]. Those findings are consistent with our data obtained from the woodchuck model, in which ETV treatment alone only induced either only transient CTL responses [91] or no responses at all [93]. Therefore, additional strategies that can potently enhance T cell response need to be enroled for the treatment of CHB infection.

Recent studies in chronic virus infection models indicate that the interaction between the inhibitory receptor programmed death-1 (PD-1) and its ligands plays a critical role in T cell exhaustion [94–97]. In chronic HBV infections, upregulation of PD-1 on virus-specific T cells was observed, and restoration of the T cell function has been achieved by blocking the PD-1/PD-ligand 1 (PD-L1) interaction in vitro [98]. Recently, the therapeutic effect of PD-1/PD-L1 blockade has also been investigated for chronic HCV infection in chimpanzees [99] and in patients [100]. However, limited effect on restoring T cell function was observed in these studies which used only PD-1/PD-L1 blockade. It has been recently clarified that the proportion of CD8 T cells expressing PD-1 and the levels of PD-1 on virus-specific T cells are strongly correlated with viral load in the plasma [101–103]. Antiretroviral treatment resulted in the dramatic decline of plasma viral load, coincident with a decrease in the PD-1 expression level on virus-specific CD8 T cells [101, 103]. In line with this, a better restoration of T cell functions upon in vitro anti-PD-L1 treatment was observed in chronic HBV patients with lower viremia [104]. Therefore, a combination therapy that includes direct antiviral drug and PD-L1 blockade is a reasonable strategy for the treatment of chronic HBV infection.

In line with these findings, Zhang et al. [105] and Liu et al. [93] successfully cloned and characterized the woodchuck PD-1/PD-L system in the WHV infection woodchuck model. A significant positive correlation between the viral load and the PD-1 expression on total CD8 T cells in chronic WHV infection was observed. Both the proportion of PD-1+ CD8 T cells and the levels of PD-1 expression on CD8 T cells were significantly higher in the woodchucks with chronic WHV infection compared to naïve animals and resolvers. More importantly, during ETV treatment of those chronic carriers, a reduction of serum viral load was correlated with a dramatic decrease in the level of PD-1 expression on CD8 T cells [93]. In vitro blockade of woodchuck PD-1/PD-L1 pathway by using a rabbit polyclonal PD-L1 blocking antibody could partially restore the T cell function in WHV-infected woodchucks [105]. Moreover, in vivo blockade of the PD-1/PD-L1 pathway on CD8 T cells, in combination with nucleoside analogue treatment and DNA vaccination, synergistically enhanced the function of virus-specific T cells. The combination therapy potently suppressed WHV replication, leading to sustained immunological control of viral infection, anti-WHs antibody development and complete viral clearance in some woodchucks [93]. Although similar approaches have been tried in other viruses in the past, such as LCMV, the data presented here may be an advance for the HBV field to new approaches for eliminating the virus itself rather than only suppressing its replication.

## Summary and conclusion

**Fig. 2:**
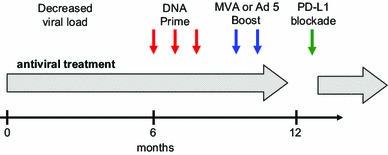
Potential schemes for immunotherapy of patients with chronic hepatitis B. Ideally, patients should be already HBV DNA negative under antiviral treatment, have seroconverted to anti-HBe, have low or moderate HBsAg concentrations and normal or only slightly elevated ALT at beginning of vaccination or/and PDL-1 antibody treatment. Three options for combination with nucleot(s)ide analogues therapy are suggested: (1) PDL-1 blockade; (2) prime-boost vaccination; (3) prime-boost vaccination and subsequent PDL-1 blockade

The woodchuck is a valuable preclinical model for developing new therapeutic approaches in chronic hepadnaviral infections. Even though several innovative approaches combining antiviral treatment with nucleoside analogues, DNA vaccines and protein vaccines were tested in chronically infected woodchucks, the effectiveness of those strategies was very limited. Strategies investigated so far were often hampered by weak T cell responses observed after immunization, suggesting a strong need for alternative strategies to enhance T cell functions during chronic HBV infection. Recently, our group published two independent proof-of-concept studies, showing that using a very potent T cell vaccine and blockade of negative signalling in T cells may lead to the resolution of chronic hepatitis B in some woodchucks (Table 3). These data are encouraging and implicate the feasibility and usefulness of the immunotherapeutic strategies for the treatment of chronically HBV-infected patients. Nevertheless, which factors influence the effect of therapeutic vaccination remains to be further investigated. It has been noticed that satisfactory therapeutic effects could not be documented in the studies using HBsAg-based prophylactic vaccines. In the mean time, evidence has supported that HBcAg-specific immunity is endowed with antiviral and liver-protecting capacities in CHB patients and animal models. With the increasing number of available vaccine formulation, a more crucial question raised recently: what is the optimal combination of these vaccines. Obviously, it is necessary to test the mutual influences of different types of vaccines to maximize their effects and avoid the negative interference between the vaccines. Also, the question how HBV infection leads to defective immune responses to HBV proteins remains to be investigated. This issue is the key to a more rational design of new therapeutic approaches. Figure 2 summarizes the ideas of a potential combination treatment for patients with chronic hepatitis B.

**Table 3:** Summary of four preclinical studies of combination therapy with entecavir and T cell vaccine performed in woodchucks

Study no. [reference]	Number of treated animals	Antiviral treatment	Duration months	Vaccines	Number of shots	Outcome	
						Delayed rebound	WHV DNA neg. in follow-up
1. Lu et al. (unpublished)	9	ETV 0.5 mg/kg	6	DNA vaccine WHsAg, WHcAg	6	9/9	1/7 (14.3 %)
2. Lu et al. (unpublished)	6	ETV 0.2 mg	12	DNA vaccine WHsAg, WHcAg	12	6/6	2/6 (33.3 %)
3. [91]	4	ETV 0.2 mg/kg	6	DNA and AdV vaccine WHcAg	9	4/4	2/4 (50.0 %)
4. [93]	3	ETV 0.2 mg/kg	6	DNA vaccine WHsAg, WHcAg Anti-PDL1	12	3/3	2/3 (66.7 %)
Total	22 vaccinated animals Ten control animals in four studies					22/22 0/10	7/20 (35.0 %) 0/10

## Unresolved problems

The presence of viral components may be a main reason for T cell tolerance in chronic HBV infection. Antiviral treatment with nucleoside analogues efficiently reduce HBV replication and release of new virions and may partly restore HBV-specific CD8 and CD4 T cell responses, thereby allowing successful therapeutic vaccination. However, HBV proteins are still produced as the transcription of mRNAs for the S protein and the core protein on HBV cccDNA is not affected by antiviral treatment. Even when HBV DNA disappears during antiviral treatment, HBsAg and HBcAg/HBeAg are present in the liver or in blood at high levels. It is proposed to reduce HBV protein load by small interfering RNAs (siRNAs), which lead to the sequence-specific degradation of homologous mRNA. Using this RNA interference (RNAi) with chemically synthesized or vector-expressed siRNAs, many clinically relevant viruses including the human immunodeficiency virus, HBV and HCV could be inhibited. In in vitro experiments showed that WHV transcripts could be degraded by siRNAs [106]. At the same time, the degradation of viral RNAs resulted in the activation of multiple pathways of host innate immune responses [107]. However, future in vivo studies are required to demonstrate the usefulness of this technology. Combining gene-silencing approach with nucleoside analogues may further facilitate the stimulation of the immune system by therapeutic vaccines.

The epigenetic regulation provides an alternative to interfere with HBV gene expression. HBV minichromosome in hepatocytes is under the complex control of epigenetic mechanisms, and its transcriptional activity could be influenced by methylation, histone acetylation and other mechanisms [108]. Therefore, exploring epigenetic drugs to modify, these regulatory processes may achieve an effective suppression of HBV gene expression and thereby replace antiviral treatment with nucleoside analogues.

The stimulation of innate immune responses may contribute to the control of HBV infection. In this special issue, Zhang and Lu provided a review dedicating to the role of TLR system. Interferons and interferon-stimulated genes (ISGs) represent still an important part for anti-HBV treatment. A recent review about this aspect described the current progress (Pei et al., in press). Recently, the antiviral functions of ISGs are under studies. For example, interferon-induced protein with tetratricopeptide repeats 1 and 2 is a cellular factor that was shown to limit hepatitis B virus replication in hepatoma cells [109]. Another recent report by Lucifora et al. [70] about the role of APOBECs in the degradation of cccDNA was highly interesting, but remained to be controversial [110, 111]. Future investigation is required to elucidate the functions of ISGs and their relative contribution for control of HBV infection, before exploring these genes for antiviral treatment.
